# Supplementary material for: Occurrence and Risk Assessment of Personal PM2.5-Bound Phthalates Exposure for Adults in Hong Kong
Source: Int J Environ Res Public Health. 2022 Oct 18;19(20):13425. doi: 10.3390/ijerph192013425 (PMC9602720; doi:10.3390/ijerph192013425)
Supplement: Supplementary file 1 [file ijerph-19-13425-s001.zip › ijerph-1930070-supplementary.pdf]

Supplementary Materials

Occurrence and Risk Assessment of Personal $PM_{2.5}$-Bound Phthalates Exposure for Adults in Hong Kong

Jiayao Chen [1,2,*], Tony J. Ward [3], Steven Sai Hang Ho [4] and Kin Fai Ho [5]

1 Department of Real Estate and Construction, The University of Hong Kong, Hong Kong Special Administrative Region, China

2 Shenzhen Institute of Research and Innovation, The University of Hong Kong, Shenzhen 518057, China

3 School of Public and Community Health Sciences, University of Montana, Missoula, MT 59801, USA

4 Division of Atmospheric Sciences, Desert Research Institute, Reno, NV 89512, USA

5 The Jockey Club School of Public Health and Primary Care, The Chinese University of Hong Kong, Hong Kong Special Administrative Region, China

* Correspondence: chenxcui@hku.hk

Content

Text

S1. Search strategy
S1.1. Search Strategy for Web of Science
#1 TS= “phthalate*” OR “phthalate ester*” OR “phthalic acid ester” OR “endocrine disruptor*” OR “endocrine disrupting chemical*” OR “phthalate congener*”
#2 TS= “fine particle” OR “fine particulate matter” OR PM2.5 OR PM 2.5 OR PM25 OR PM 25
3 TS= “personal exposure” OR “individual exposure” OR “indoor*” OR “residential indoor*” OR outdoor OR ambient
Search 4: #1 AND #2 AND #3
Results = 88

S1.2. Search Strategy for PubMed

Search	Actions	Details	Query	Results	Time
#4			Search: #1 AND #2 AND #3	38	01:38:13
#3			Search: "personal exposure" OR "individual exposure" OR "indoor*" OR "residential indoor*" OR outdoor OR ambient	141,722	01:37:49
#2			Search: "fine particle" OR "fine particulate matter" OR PM2.5 OR PM 2.5 OR PM25 OR PM 25	29,017	01:37:25
#1			Search: ("phthalate*" OR "phthalate ester*" OR "phthalic acid ester" OR "endocrine disruptor*" OR "endocrine disrupting chemical*" OR "phthalate congener*") OR ("phthalate*" OR "phthalate ester*" OR "phthalic acid ester" OR "endocrine disruptor*" OR "endocrine disrupting chemical*" OR "phthalate congener*")	25,141	01:37:07

S1.3. Search Strategy for Scopus

(TITLE-ABS-KEY ("phthalate*" OR "phthalate ester*" OR "phthalic acid ester" OR "endocrine disruptor*" OR "endocrine disrupting chemical*" OR "phthalate congener*")) AND (TITLE-ABS-KEY ("fine particle" OR "fine particulate matter" OR pm2.5 OR pm 2.5 OR pm25 OR pm 25)) AND (TITLE-ABS-KEY ("personal exposure" OR "individual exposure" OR "indoor*" OR "residential indoor*" OR outdoor OR ambient))

Results = 6

S2. Estimation of PAEs exposure using time-activity weighted model

A time-activity weighted model was used to estimate total personal exposure to PAE congener and mixtures in adults using the following equation:

$$\text{Estimated exposure} = \sum_{k=1}^{n} C_{ik} t_{ijk} / T_{ij} \qquad \text{(S1)}$$

where C_{ik} represents the PAE_i exposure concentration in microenvironment k (home, office/school, outdoors in Table Sx) for subject i, and t_{ijk} refers to time (min) in microenvironment k for subject i on sampling day j. T_{ij} is the total sampling time (1440 min).

Chen et al. (2018) incorporated the time indoors and outdoors to estimate daily intake of PAEs via inhalation. In the current study, residential indoor and outdoor PAEs were directly measured; workplace and school PAE concentrations were estimated based on personal PAE concentrations and the corresponding time fraction. A Monte Carlo simulation was employed to estimate the distribution of PAE exposures and health risks attributable to DEHP inhalation exposure for adults.

Figures:

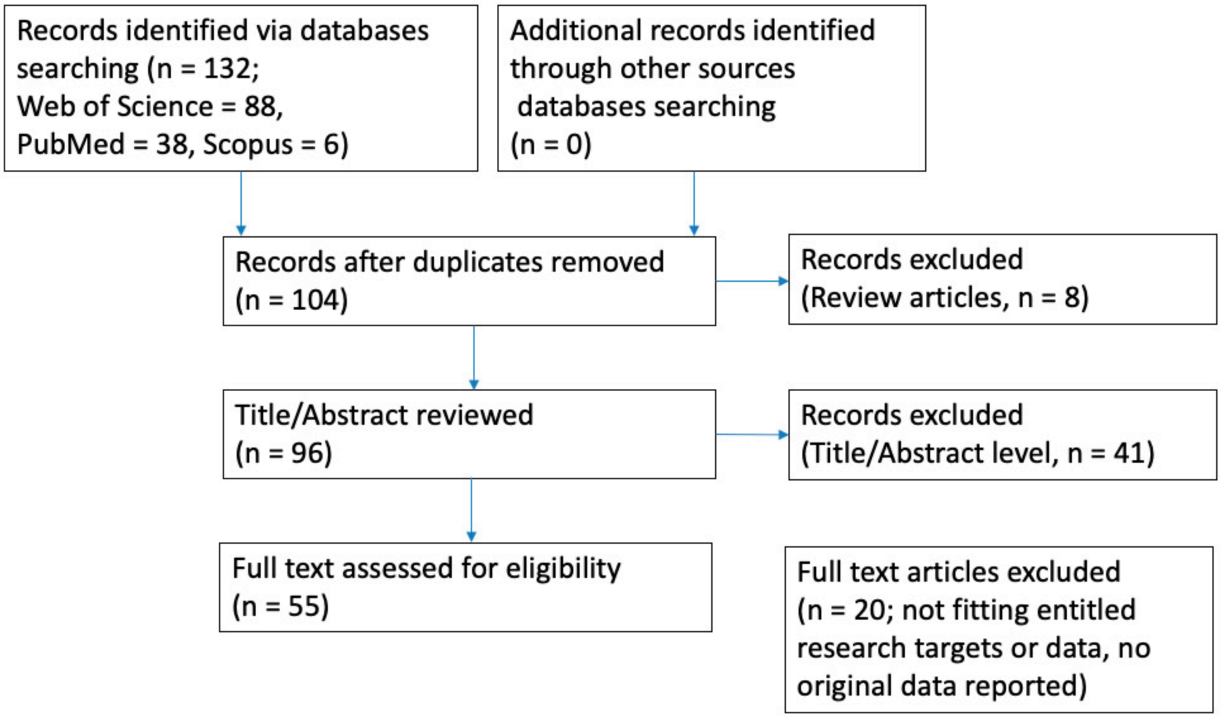

Figure S1. PRISMA flow diagram.

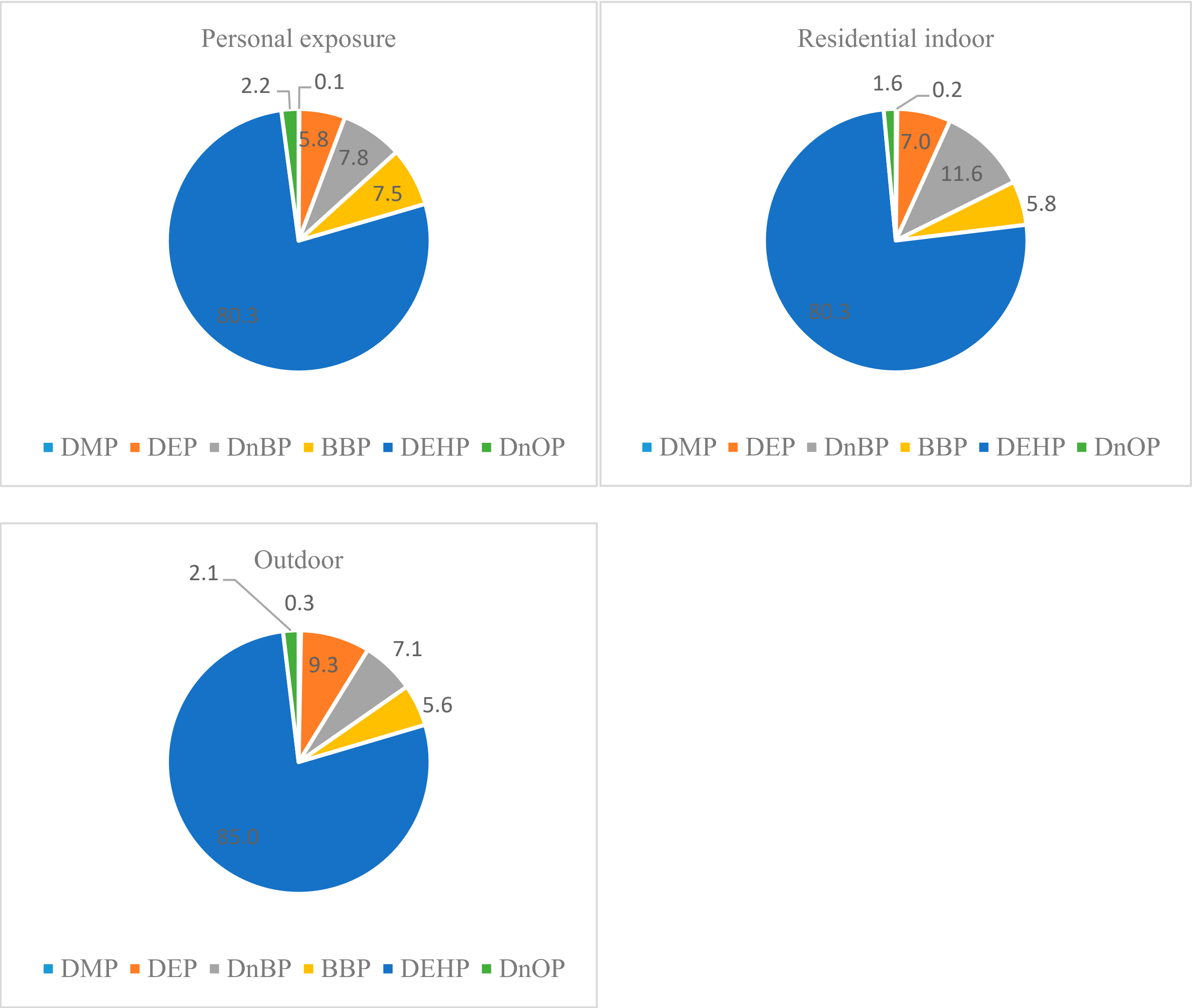

Figure S2. Percentage contribution of individual PAE congener to $\sum_6$PAEs concentrations in personal, residential indoor, and outdoor $PM_{2.5}$.

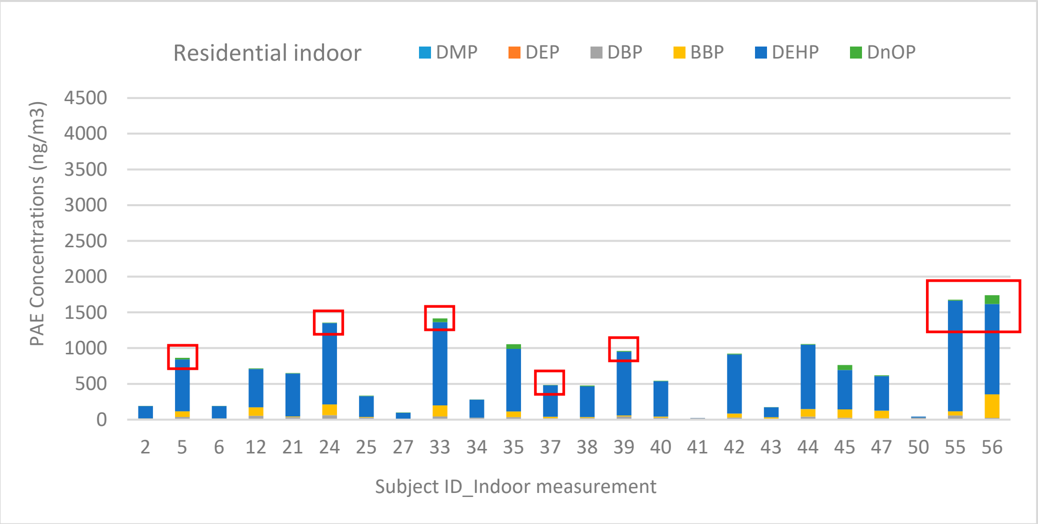
Residential indoor
DMP
DEP
DBP
BBP
DEHP
DnOP
PAE Concentrations (ng/m3)
4500
4000
3500
3000
2500
2000
1500
1000
500
0
2 5 6 12 21 24 25 27 33 34 35 37 38 39 40 41 42 43 44 45 47 50 55 56
Subject ID_Indoor measurement

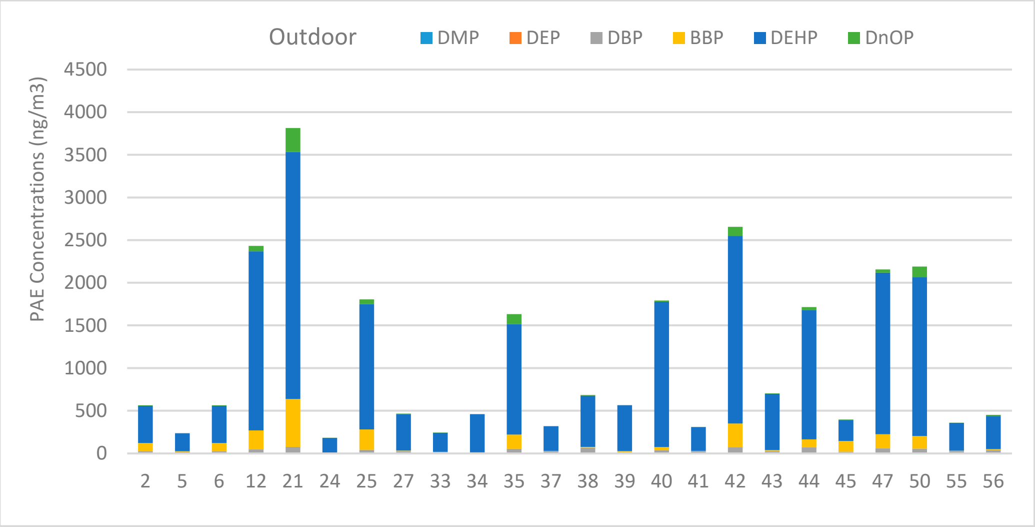
Outdoor
DMP
DEP
DBP
BBP
DEHP
DnOP
PAE Concentrations (ng/m3)
4500
4000
3500
3000
2500
2000
1500
1000
500
0
2 5 6 12 21 24 25 27 33 34 35 37 38 39 40 41 42 43 44 45 47 50 55 56

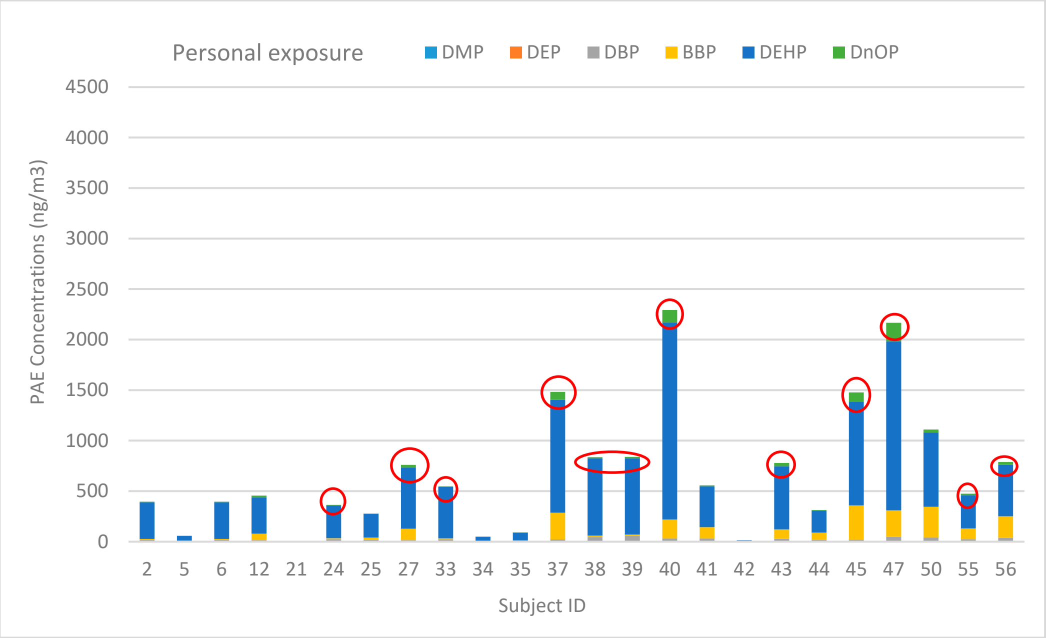
Personal exposure
DMP
DEP
DBP
BBP
DEHP
DnOP
PAE Concentrations (ng/m3)
4500
4000
3500
3000
2500
2000
1500
1000
500
0
2 5 6 12 21 24 25 27 33 34 35 37 38 39 40 41 42 43 44 45 47 50 55 56
Subject ID

Figure S3. The $\sum_6$PAEs concentrations and PAE congener distribution characteristics (ng/m^3) in concurrent residential indoor, outdoor, and personal $PM_{2.5}$.

Table S1. Location and description of ambient sampling site.

District	Location of ambient sampling site	Sampling date	Characteristics
Central and Western District, Hong Kong Island	~ 15 m above ground, Patrick Manson Building at the School of Public Health, The University of Hong Kong (HKU) (22.27° N, 114.13°E)	June–Sep 2014; Dec 2014–March 2015; Aug–Sep 2015; March 2016	The ambient sampling site was located in a mixed residential/commercial area, with recreational areas and open green spaces.
Kowloon City, Kowloon	~ 25 m above ground, Industrial Centre Building at the Hong Kong Polytechnic University (HKPU) (22.31° N, 114.18°E)	July–Oct 2014; Nov 2014–Jan 2015; Aug 2015; Jan–March 2016	Densely populated residential inner-city district with busy traffic (e.g., eight meters away from the main traffic road) and commercial activities.
Sha Tin District, New Territories	~ 20 m above ground, Shaw Auditorium at Prince of Wales Hospital, The Chinese University of Hong Kong (CUHK) (22.38° N, 114.20°E)	July–Oct 2014; Oct–Dec 2014; Aug 2015; Jan-Feb 2015	A district with mixed land-use patterns with some wide roads nearby and commercial activities.

Table S2. Method detection limits (MDLs) and estimated exposure frequency of the studied phthalate esters across different exposure categories.

		> MDLs			
Phthalate eaters (Abbreviation)	MDLs (ng/filter)	Personal	Residential indoor	Outdoor	Average
dimethyl phthalate (DMP)	0.04	93.9%	95.4%	96.7%	95.3%
diethyl phthalate (DEP)	2.63	97.2%	96.9%	100.0%	98.1%
di-n-butyl phthalate (DnBP)	2.18	99.4%	100.0%	100.0%	99.8%
butyl benzyl phthalate (BBP)	0.42	91.2%	84.6%	85.0%	86.9%
di(2-ethylhexyl)phthalate (DEHP)	0.47	96.7%	93.8%	93.3%	94.6%
di-n-octyl phthalate (DnOP)	n.d.[a]	84.5%	78.5%	85.0%	82.7%
Average frequency of detection		93.8%	91.5%	93.3%	
Estimated exposure frequency (days/year)		342	334	341	

Note: [a]n.d.: not detectable.

[b] Estimated exposure frequency = Average frequency of detection of PAEs * 356 days/year.

Table S3. Spatial variation of PAE congeners (mean value) and PAE mixtures (ng/m^3) in outdoor $PM_{2.5}$.

Species	HKU	HKPU	CUHK	*p*-value[b]
DMP	0.16	0.18	0.14	0.57
DEP	3.77	2.84	3.56	0.48
DnBP	43.9	32.9	28.0	0.30
BBP	123.4	118.8	117.2	0.99
DEHP	896.4	1177.9	893.5	0.74
DnOP	38.7	46.3	65.8	0.67
$\sum_6$PAEs [a]	1096.2	1181.0	1083.7	0.98

Notes: [a] $\sum_6$PAEs: the sum of six PAE congeners, including DMP, DEP, DnBP, BBP, DEHP, and DnOP.

[b] A *p*-value < 0.05 indicates significant spatial variation.

Table S4. Reported concentrations of particle-phase PAEs in ambient $PM_{2.5}$ collected from various study areas (unit: ng/m^3).

Site (Study area)	Season	DMP	DEP	DnBP	BBP	DEHP	DnOP	Mean (SD)	Range (Min–Max)	References
Tibetan Plateau, China	Summer	—	—	++	—	++	—	1.9 (1.2)	0.3–5.5	Jun Li et al. (2013)
16 urban sites, China	Summer/Winter	nd–5.7	nd–23	14–269	nd–8.7	25–856	—	nr	63–1162	Li andWang (2015)
Beijing, China	Summer	++	++	++	—	++	—	387 (137)	178–631	Yang et al. (2016)
Nanjing, China	Mixed	1.1	0.4	10	0.6	4.6	0.3	1.7 $^{\$}$	nr	Wang et al. (2008)
Nanjing, China		0.3	0.2	3.1	0.2	1.2	0.2	5.2 $^{\$}$	nr	
Background site-Mount Tai, China	Summer	0.2	1	1.6	0.1	12.5	0.5	15.8	nr	Li et al. (2019)
Guangzhou, China	Summer	18.5	8.6	162	32.6	21.6	42.3	285.6 $^{\$}$	nr	Wang et al. (2017)
Guangzhou, China	Winter	23.8	13	408	39.5	148	202	**834.3** $^{\$}$	nr	
Beijing, China	Winter	19.1	3.1	5.2	nd	28.7	nd	55.6	nr	Li et al. (2022)
Xi'an, China	Winter	41.4–98.4	63.8–93.6	28.5–167.2	85.8–207.8	147.9–875.8	71.4–118.3	nr	nr	Wang et al. (2018)
IG, India	Summer/Winter	3.2	2.4	4.3	1.9	24.1	32.3	68.3	nr	Gadi et al. (2018)
MN, India		2.9	2.5	2.3	2.4	27.5	32.7	70.4	nr	
HR, India		3.6	2.6	4.3	2.2	32.2	19.6	64.5	nr	
Japan	Mixed	—	—	—	0.19	—	—	na	na	Ichikawa et al. (2018)
Shanghai, China	Mixed-Summer and Winter	0.32	0.19	6.72	0.47	39.5	—	47.2$^{\$}$	na	Ma et al. (2014)
Four sites in Tianjin, China	Summer	0.19	0.11	3.2	0.03	19.7	0.18	23.4	9.4–54.9	Zhu et al. (2016)
Four sites in Tianjin, China	Winter	0.14	0.05	1.3	0.005	2.6	0.01	**4.2**	1.7–9.8	
Urban Xian, China	Mixed	3.95	0.65	15.5	9.78	54.8	0.79	85.5	2.5–414	Ma et al. (2020)
Background, Mount Tai (top), China	Mixed	3.78	0.74	76.8	nd	38.8	0.07	120.2	nr	Zhen et al. (2019)
Urban area, Mount Tai (foot), China		0.26	0.74	107.3	nd	21.3	0.88	130.4	nr	
Mexico City		—	—	nd	7.2	671.5	—	na	na	Quintana-Belmares et al. (2018)
Hangzhou, China	Mixed	0.42	0.61	3.3	1.7	113	0.47	119.5$^{\$}$	nr	Lu andZhu (2021)
IG, India	Mixed	10.7	45.2	51	30.5	94.4	11	210.8$^{\$}$	nr	Gadi et al. (2019)
MN, India		6.2	15.5	48.7	15.2	93.7	14.6	158.9$^{\$}$	nr	
HR, India		5.1	13.1	22.7	15.1	91.5	31.7	130.4$^{\$}$	nr	
Shenzhen, China	Mixed-Summer and Winter	—	1.64	2.88	—	59.9	5.12	65.9$^{\$}$	na	Lu et al. (2018)
Guangzhou, China		0.16	nd	28.2	—	30.7	—	59.1$^{\$}$	na	Zhang et al. (2019)
Shanghai, China		0.06	0.02	9.26	—	41.5	—	50.8$^{\$}$	na	
Beijing, China		0.37	0.06	9.17	—	34.2	—	43.8$^{\$}$	na	
Harbin, China		2.21	0.14	40.1	—	93.8	—	136.3$^{\$}$	na	
Tianjin, China	Mixed	0.54	0.3	8.72	0.08	75.7	0.33	85.7$^{\$}$	na	Kong et al. (2013)

Notes: ++: Analyzed, but no specific values were reported; —: Not analysed; nr: Not reported; nd: Not detected; < MDL: blow method detection limit; mean: average value of summation of six PAE congeners; SD: Standard deviation; range: Minimum-Maximum. $^{\$}$ Calculated based on individual PAE concentrations.

Table S5. PAE congener concentrations in matched personal exposure, residential indoor, and outdoor $PM_{2.5}$ in summer and winter season.

ng/m^3	Personal					Indoor					Outdoor				
	Summer		Winter			Summer		Winter			Summer		Winter		
	Mean	SD[a]	Mean	SD	*p*-value[c]	Mean	SD	Mean	SD	*p*-value	Mean	SD	Mean	SD	*p*-value[c]
DMP	0.10	0.08	0.10	0.07	0.90	0.15	0.14	0.19	0.20	0.29	0.14	0.07	0.19	0.15	0.05
DEP	1.90	1.52	2.30	3.15	0.54	2.57	2.24	3.87	3.65	0.10	2.62	1.71	3.87	2.76	0.04
DnBP	18.2	20.3	23.2	21.3	0.36	23.7	16.7	30.4	30.8	0.29	36.9	34.4	31.5	29	0.50
BBP	54.4	107.2	133.8	179.4	0.05	47.1	88.8	83.2	147.6	0.28	143.9	209.7	93.1	181.1	0.36
DEHP	488.6	719.5	667.5	720.4	0.35	471.6	404.3	696.6	749.5	0.16	1140.7	1198.4	887.8	1063	0.41
DnOP	25.9	63.2	38.9	76.2	0.50	8.5	10.8	32.5	68.7	0.10	65.1	101.7	32.3	71.3	0.19
$\sum_6$PAEs[b]	562.1	862.5	832.0	929.7	0.25	529.1	453.2	761.1	922.9	0.21	1242.6	1460.7	973.6	1260	0.45

Notes*:* [a] SD refers to standard deviation.

[b] $\sum_6$PAEs: the sum of six PAE congeners, including DMP, DEP, DnBP, BBP, DEHP, and DnOP.

[c] A *p*-value < 0.05 indicates significant seasonal variation.

Table S6. Inhalation rate (m^3/day) of different activities for male and female adult participants (ages 18 – 42).

	Activities [a]	Adult females	Adult males
	Lying (m^3/min)	0.00712	0.00893
	Sitting (m^3/min)	0.00772	0.0093
	Standing (m^3/min)	0.00836	0.01065
	Walking (m^3/min) (2.25 mph)	0.0203	0.0241
	Running (m^3/min) (4.5 mph)	0.0479	0.0573
	Car driving (m^3/min)	0.00895	0.0108
	Car riding (m^3/min)	0.00819	0.00983
	Housework (m^3/min)	0.0174	/[b]
Collectively	Resting (m^3/h)	0.36	0.45
	Light activities (m^3/h)	1.14	1.2
	Moderate activities (m^3/h)	/	/
Inhalation rate (m^3/day) [c]	Mean ± standard deviation	15.4 ± 2.1	14.9 ± 2.1
	95th percentile	20.7	22.4
Weight (kg)	Mean ± standard deviation	55.2 ± 5.0	55.2 ± 11.2

Notes: [a] Data source: Exposure Factors Handbook (Table 6-5, Table 6-26, Table 6-40, Table 6-41), U.S. EPA, September 2011.

[b] Not available.

[c] Exposure Factor handbooks of the U.S. EPA reported the long-term inhalation rates for 16-51 ages were 16.0 and 22.1 m^3/day, respectively.

Table S7. Time activities for subjects involved in concurrent measurements

Time-activity data from diaries (n = 55)	Mean (standard deviation) (min)			
**Indoors**	Summer	Winter	*p*-value	Total
Indoors, total	1056 (529)	1186 (461)	0.30	1119 (497)
Indoors, at home	1053 (310)	1099 (289)	0.57	1076 (297)
Indoors, office/school	176 (250	213 (248)	0.59	195 (247)
**Outdoors**	351 (288)	434 (255)	0.29	388 (274)

Table S8. Measure and estimated $PM_{2.5}$-bound PAEs for study participants

Personal exposure	Measured				Modelled				
Exposure Con. (ng/m^3)	Mean	SD[a]	Median	95th[c]	Mean	SD	Median	95th	*p*-value[d]
DMP	0.10	0.07	0.08	0.22	0.16	0.15	0.12	0.40	0.01
DEP	2.05	2.42	1.53	6.04	3.20	2.84	2.34	8.46	0.02
DnBP	20.65	20.61	13.68	62.74	29.48	24.25	23.08	67.02	0.04
BBP	87.14	139.52	7.21	359.97	60.19	98.19	24.82	237.34	0.26
DEHP	560.48	695.45	377.42	2352.74	660.00	606.56	407.15	1845.73	0.43
DnOP	27.81	59.26	5.69	165.36	20.25	40.77	3.96	87.43	0.45
$\sum_6$PAEs[d]	655.30	843.20	341.35	2864.44	724.10	729.66	425.15	2098.34	0.64

Notes: [a] SD: Standard deviation.

[c] $\sum_6$PAEs: summation of the six PAE congeners.

[c] 95th: The 95th percentile.

[d] A *p*-value < 0.05 indicates significant difference between measured and estimated results.

Table S9. Exposure concentration (ng/m^3) and daily intake (DI_{inh}) of PAE congeners (µg/kg-day) via inhalation route for female and male adult participants.

	Female				Male				
Exposure Con. (ng/m^3)	Mean	SD [a]	Median	95th	Mean	SD	Median	95th	*p*-value [c]
DMP	0.09	0.08	0.07	0.23	0.11	0.09	0.09	0.29	0.10
DEP	1.99	1.94	1.51	6.26	2.00	2.23	1.44	5.55	0.96
DnBP	19.31	20.09	12.65	52.86	21.12	20.79	14.82	69.24	0.56
BBP	70.82	106.94	16.59	255.50	99.76	150.87	9.98	434.51	0.15
DEHP	473.48	587.35	344.44	1244.48	634.80	823.24	324.02	2379.36	0.13
DnOP	19.42	50.11	5.07	69.27	50.91	119.88	7.00	215.83	0.03
$\sum_6$PAEs [d]	572.45	713.08	392.47	1402.29	765.71	1019.67	366.43	2877.61	0.14
DI_{inh} (µg/kg-day)									
DMP	2.6E-05	2.8E-05	1.9E-05	9.0E-05	3.3E-05	7.4E-05	1.8E-05	7.7E-05	0.38
DEP	5.5E-04	6.0E-04	3.7E-04	1.8E-03	5.9E-04	1.5E-03	3.1E-04	1.4E-03	0.81
DnBP	5.5E-03	6.7E-03	3.2E-03	1.7E-02	4.8E-03	5.9E-03	3.1E-03	1.5E-02	0.45
BBP	2.2E-02	3.8E-02	5.7E-03	8.5E-02	2.3E-02	3.6E-02	2.1E-03	1.0E-01	0.84
DEHP	1.4E-01	2.0E-01	9.2E-02	3.4E-01	1.6E-01	2.2E-01	7.7E-02	5.9E-01	0.65
DnOP	6.0E-03	1.5E-02	1.3E-03	1.9E-02	1.1E-02	2.2E-02	1.9E-03	6.4E-02	0.10
$\sum_6$PAEs [d]	1.7E-01	2.4E-01	1.0E-01	3.8E-01	1.8E-01	2.6E-01	8.8E-02	7.2E-01	0.68
CR_{inh} [e]									
DEHP	1.2E-06	1.5E-06	6.5E-07	4.2E-06	1.2E-06	1.5E-06	6.5E-07	4.2E-06	0.65

Notes*:* [a] SD refers to standard deviation.

[b] 95th: The 95th percentile.

[c] The level of significance was taken as *p*-value < 0.05; we did not find statistical differences of average daily intake of PAE across adult male and female subjects.

[d] $\sum_6$PAEs: summation of the six PAE congeners.

[e] An inhalation cancer potency factor of 0.0084 (mg/kg-day)$^{-1}$ was used and results were estimated using Monte Carlo Simulation.

References:

1. Chen Y, Lv D, Li X, Zhu T (2018): PM2.5-bound phthalates in indoor and outdoor air in Beijing: Seasonal distributions and human exposure via inhalation. Environ Pollut 241, 369-377
2. Elzein A, Stewart GJ, Swift SJ, Nelson BS, Crilley LR, Alam MS, Reyes-Villegas E, Gadi R, Harrison RM, Hamilton JF (2020): A comparison of PM 2.5-bound polycyclic aromatic hydrocarbons in summer Beijing (China) and Delhi (India). Atmospheric Chem. and Phys. 20, 14303-14319
3. Gadi R, Sharma SK, Mandal TK, Kumar R, Mona S, Kumar S, Kumar S (2018): Levels and sources of organic compounds in fine ambient aerosols over National Capital Region of India. Environmental Science and Pollution Research 25, 31071-31090
4. Gadi R, Sharma SK, Mandal TK (2019): Source apportionment and health risk assessment of organic constituents in fine ambient aerosols (PM2. 5): a complete year study over National Capital Region of India. Chemosphere 221, 583-596
5. Ichikawa Y, Watanabe T, Horimoto Y, Ishii K, Naito S (2018): Measurements of 50 non-polar organic compounds including polycyclic aromatic hydrocarbons, n-alkanes and phthalate esters in fine particulate matter (PM 2.5) in an industrial area of Chiba prefecture, Japan. Asian Journal of Atmospheric Environment 12, 274-288
6. Jun Li J, Hui Wang G, Ming Wang X, Ji Cao J, Sun T, Lei Cheng C, Jing Meng J, Feng Hu T, Xin Liu s (2013): Abundance, composition and source of atmospheric PM2. 5 at a remote site in the Tibetan Plateau, China. Tellus B: Chemical and Physical Meteorology 65, 20281
7. Kong S, Ji Y, Liu L, Chen L, Zhao X, Wang J, Bai Z, Sun Z (2013): Spatial and temporal variation of phthalic acid esters (PAEs) in atmospheric PM10 and PM2. 5 and the influence of ambient temperature in Tianjin, China. Atmospheric Environment 74, 199-208
8. Larsen JC, Larsen PB (1998): Chemical Carcinogens. In: Hester RE , Harrison RM (Editors), Air Pollution and Health. The Royal Society of Chemistry, pp. 33-56
9. Li J, Wang G (2015): Airborne particulate endocrine disrupting compounds in China: Compositions, size distributions and seasonal variations of phthalate esters and bisphenol A. Atmospheric Research 154, 138-145
10. Li P-h, Jia H-y, Wang Y, Li T, Wang L, Li Q-q, Yang M-m, Yue J-j, Yi X-l, Guo L-q (2019): Characterization of PM2. 5-bound phthalic acid esters (PAEs) at regional background site in northern China: long-range transport and risk assessment. Science of The Total Environment 659, 140-149
11. Li X, An Z, Shen Y, Yuan Y, Duan F, Jiang J (2022): Dynamic variations of phthalate esters in PM2. 5 during a pollution episode. Science of The Total Environment 810, 152269
12. Lu H, Zhu Z (2021): Pollution characteristics, sources, and health risk of atmospheric phthalate esters in a multi-function area of Hangzhou, China. Environ Sci Pollut Res Int 28, 8615-8625

13. Lu S, Kang L, Liao S, Ma S, Zhou L, Chen D, Yu Y (2018): Phthalates in PM2. 5 from Shenzhen, China and human exposure assessment factored their bioaccessibility in lung. Chemosphere 202, 726-732
14. Ma B, Wang L, Tao W, Liu M, Zhang P, Zhang S, Li X, Lu X (2020): Phthalate esters in atmospheric PM2.5 and PM10 in the semi-arid city of Xi'an, Northwest China: Pollution characteristics, sources, health risks, and relationships with meteorological factors. Chemosphere 242, 125226
15. Ma J, Chen L-l, Guo Y, Wu Q, Yang M, Wu M-h, Kannan K (2014): Phthalate diesters in Airborne PM2. 5 and PM10 in a suburban area of Shanghai: Seasonal distribution and risk assessment. Science of the total environment 497, 467-474
16. Nisbet IC, Lagoy PK (1992): Toxic equivalency factors (TEFs) for polycyclic aromatic hydrocarbons (PAHs). Regulatory toxicology and pharmacology 16, 290-300
17. OEHHA (2009): Technical Support Document for Cancer Potency Factors 2009: Appendix H. Exposure routes and studies used to derive cancer unit risks and slope factors. . California Office of Environmental Health Hazard Assessment, California Environmental Protection Agency
18. Quintana-Belmares RO, Krais AM, Esfahani BK, Rosas-Pérez I, Mucs D, López-Marure R, Bergman Å, Alfaro-Moreno E (2018): Phthalate esters on urban airborne particles: levels in PM10 and PM2. 5 from Mexico City and theoretical assessment of lung exposure. Environmental research 161, 439-445
19. Wang J, Guinot B, Dong Z, Li X, Xu H, Xiao S, Ho SSH, Liu S, Cao J (2017): PM2. 5-bound polycyclic aromatic hydrocarbons (PAHs), oxygenated-PAHs and phthalate esters (PAEs) inside and outside middle school classrooms in Xi'an, China: Concentration, characteristics and health risk assessment. Aerosol and Air Quality Research 17, 1811-1824
20. Wang J, Dong Z, Li X, Gao M, Ho SSH, Wang G, Xiao S, Cao J (2018): Intra-urban levels, spatial variability, possible sources and health risks of PM2. 5 bound phthalate esters in Xi'an. Aerosol and air quality research 18, 485-496
21. Wang P, Wang S, Fan C (2008): Atmospheric distribution of particulate-and gas-phase phthalic esters (PAEs) in a Metropolitan City, Nanjing, East China. Chemosphere 72, 1567-1572
22. Yang F, Kawamura K, Chen J, Ho K, Lee S, Gao Y, Cui L, Wang T, Fu P (2016): Anthropogenic and biogenic organic compounds in summertime fine aerosols (PM2. 5) in Beijing, China. Atmospheric Environment 124, 166-175
23. Zhang X, Wang Q, Qiu T, Tang S, Li J, Giesy JP, Zhu Y, Hu X, Xu D (2019): PM2.5 bound phthalates in four metropolitan cities of China: Concentration, seasonal pattern and health risk via inhalation. Sci Total Environ 696, 133982
24. Zhen Z, Yin Y, Chen K, Zhang X, Kuang X, Jiang H, Wang H, Cui Y, He C, Ezekiel AO (2019): Phthalate esters in atmospheric PM2. 5 at Mount Tai, north China plain: Concentrations and sources in the background and urban area. Atmospheric Environment 213, 505-514
25. Zhu Z, Ji Y, Zhang S, Zhao J, Zhao J (2016): Phthalate ester concentrations, sources,

and risks in the ambient air of Tianjin, China. Aerosol and Air Quality Research 16, 2294-2301
